# Parents' relationship with their surrogate in cross-border and domestic surrogacy arrangements: comparisons by sexual orientation and location

**DOI:** 10.1016/j.fertnstert.2018.11.029

**Published:** 2019-03

**Authors:** Vasanti Jadva, Natalie Gamble, Helen Prosser, Susan Imrie

**Affiliations:** a Centre for Family Research, University of Cambridge, Cambridge, United Kingdom; b NGA Law and Brilliant Beginnings, London, United Kingdom

**Keywords:** Surrogacy, cross-border, gay couples, heterosexual couples

## Abstract

**Objective:**

To study heterosexual and gay couples' relationship with their surrogate and their disclosure decisions when the surrogacy arrangement was completed domestically compared with internationally.

**Design:**

Cross-sectional study.

**Setting:**

Not applicable.

**Patient(s):**

Participants were 40 gay couples and 76 heterosexual couples who had domestic surrogacy in the United Kingdom (UK) (n = 38) or international surrogacy in the United States (n = 58) or Asia (20). Most (75%) of the children were aged <4 years.

**Intervention(s):**

Online surveys containing open-ended and multiple-choice questions.

**Main Outcome Measure(s):**

Experiences of finding a surrogate, relationship with the surrogate, and disclosure to the child were examined among UK parents who had undergone surrogacy in the UK, United States, or India/Thailand.

**Result(s):**

Parents who had surrogacy in the UK and United States felt very involved in the pregnancy compared with those who had surrogacy in Asia. Couples whose surrogacy was completed in Asia were less likely to want contact with their surrogate after the birth and were also less likely to have any current contact with the surrogate. Parents who had surrogacy in the UK and United States described positive relationships with their surrogate. Gay couples intended to tell their child about surrogacy more than heterosexual couples.

**Conclusion(s):**

The specific country where couples conducted their surrogacy arrangement (i.e. United States, UK, or Thailand/India) was associated with how involved they were in the pregnancy and their contact with the surrogate over time. Limitations of the study include use of survey methodology and that the representativeness of the sample is not known.

## Abstract (es)

Relación entre los padres y su subrogada tanto en los acuerdos de subrogación transfronteriza como nacional: comparación por orientación sexual y localización

**Objetivo:**

Estudiar la relación de las parejas heterosexuales y homosexuales con sus subrogadas y sus preocupaciones cuando el acuerdo de subrogación fue completado nacional frente al internacional.

**Diseño:**

Estudio transversal.

**Lugar:**

No aplicable.

**Paciente(s):**

Los participantes fuero 40 parejas homosexuales y 76 parejas heterosexuales que hicieron subrogación nacional en Reino Unido (UK) (n=38) o subrogación internacional en los Estados Unidos (n=58) o Asia (20). La mayoría de los niños fueron menores de 4 años.

**Intervención (es):**

Encuestas en línea que contenían preguntas abiertas y de opción múltiple.

**Principales medidas de resultado(s):**

Experiencias de encontrar una subrogada, relación con la subrogada y se examinó el hecho de revelárselo al niño entre los padres del Reino Unido que habían recurrido a la subrogación en el Reino Unido, Estados Unidos o India / Tailandia.

**Resultado (s):**

Los padres que hicieron la subrogación en UK y en Estados Unidos se sintieron muy involucrados en la gestación comparados con aquellos que hicieron la subrogación en Asia. Aquellas parejas cuya subrogación fue realizada completamente en Asia eran menos propensos a querer contactar con su subrogada después del nacimiento y también eran menos propensos a tener algún contacto en la actualidad con la subrogada. Los padres que hicieron la subrogación en UK y en los Estados Unidos describieron como positivas las relaciones con sus subrogadas. Las parejas homosexuales revelaron a sus hijos la subrogación más que las parejas heterosexuales.

**Conclusión (es):**

El país específico donde las parejas llevan a cabo el acuerdo para la subrogación (i.e. Estados Unidos, UK, o Tailandia/India) se asoció con cómo se involucraron durante la gestación y con el contacto en el tiempo con su subrogada. Las limitaciones del estudio incluyen el uso de la metodología de la encuesta y que la representatividad de la muestra es desconocida.

**Discuss:** You can discuss this article with its authors and other readers at **https://www.fertstertdialog.com/users/16110-fertility-and-sterility/posts/40114-26804**

A growing number of people are seeking surrogacy arrangements abroad, mainly owing to it being illegal in their country of residence (1). Despite surrogacy being permitted in the United Kingdom (UK), it too has seen a steady rise in individuals traveling abroad for surrogacy. The most recent statistics on parental orders (applications that transfer legal parenthood from the surrogate to the intended parents) show that, in 2016, 179 applications (51%) were made for UK surrogacy arrangements, and 161 (46%) for international surrogacy arrangements, which mainly took place in the United States (78) and India (63). Most applications were made by heterosexual couples (234), with approximately one-quarter (82) made by gay couples. These statistics only include those applying for parental orders, and thus parents who have surrogacy abroad but do not apply for a parental order are not included. Additionally, single persons using surrogacy cannot currently apply for parental orders, although this is expected to change (2). Thus it is feasible that more surrogacy arrangements may be taking place than the numbers suggest.

In domestic surrogacy cases in which the parents and surrogate live in the same country, gay couples in the United States and heterosexual couples in the UK have been reported to form close relationships with their surrogate, which may continue after the child is born 3, 4. Studies of surrogates have similarly reported how some surrogates maintain contact with the parents and child after the birth—a decision largely dependent on the strength of the relationship that develops between the surrogate and parents during the pregnancy (5).

Additional challenges might be faced during, and after, the pregnancy when the surrogate lives in a different country than the intended parents. In a study of Italian gay fathers who had used overseas surrogacy in Canada and the United States, the physical distance from the surrogate led many fathers to feel a lack of control over the pregnancy, which for some resulted in anxiety about how their surrogate was caring for the pregnancy. However, surrogates were also found to play an important role in helping fathers feel emotionally connected to the child (6). Unlike in the United States and Canada, where the intended parents are in contact with the surrogate, in countries such as India or Thailand direct contact is less common and may be complicated by language and cultural differences. In India, clinics play a key role in managing the pregnancy, and intended parents are not generally permitted to attend the birth, which can lead to feelings of dissatisfaction (7). A study of Israeli gay fathers who predominately had surrogacy in India found they felt anxious and frustrated over the distance and inability to emotionally connect with the pregnancy and fetus (8). Indian surrogates may also feel disappointment at not being able to meet the intended parents and see the child (9). Thus the diverse ways in which surrogacy is practiced in different countries may affect parent's experiences during the pregnancy and their subsequent relationship with the surrogate.

It is feasible that experiences of the surrogacy process may differ between heterosexual and gay parents. Research with heterosexual couple families found that intended parents typically turned to surrogacy after many years of failed IVF treatments (10). Similarly, it has been suggested that the emotional challenges of infertility and treatment may deplete parents’ psychological resources (11). Conversely, gay couples are more likely to come to surrogacy without a history of infertility and may be more likely to choose surrogacy as their preferred path to parenthood (3).

Surrogacy can place a number of stressors on the intended parents, who can feel anxious about whether the surrogate will hand over the baby (12). These anxieties might be greater when the surrogate is in a different country. On the other hand, working with a clinic or surrogacy agency might be more appealing than having to be directly involved with the surrogate, as is usually the case in the UK (13). To date, no studies have compared the experiences of parents who have domestic surrogacy with parents who travel overseas to destinations that may or may not allow direct contact with the surrogate. Given that contact with the surrogate can help intended parents feel involved in the pregnancy and emotionally connected to the unborn child 6, 8, and given the diverse ways in which surrogacy is practiced in different countries, it is important to understand how these different contexts affect the experiences of parents both during and after the pregnancy.

This study reports findings from a survey of UK parents who carried out surrogacy in the UK and overseas. Its objective was to examine differences and similarities in UK parents' relationships with the surrogate during the pregnancy and after the birth between gay and heterosexual couples and between couples who had surrogacy in the UK, United States and Asia.

## Materials and methods

### Procedure

Participants were recruited through NGA Law, an English family law firm specializing in surrogacy, and its sister organization, Brilliant Beginnings, a nonprofit UK surrogacy agency established in 2013 by the owners of NGA Law. Brilliant Beginnings is the only UK surrogacy organization to assist intended parents with domestic and international surrogacy arrangements.

E-mail invitations to take part in the study were sent to 1,212 individual e-mail addresses representing 776 families. Responses were collected over a 2-month period (February–March 2017) using an online survey. Seven hundred and twenty-nine (60%) of the e-mails were opened, and 203 surveys were completed, representing 26% of the families e-mailed.

Participants were asked to complete only one survey per couple, to avoid data from the same surrogacy arrangement being reported twice. For parents who had more than one child, the data relates to their first surrogacy arrangement (i.e., their eldest child born through surrogacy). Participants were either planning a surrogacy arrangement, had found a surrogate who was currently pregnant (or trying to conceive), or had completed a surrogacy arrangement. This paper focuses on data from 116 respondents who had completed their surrogacy arrangement in the United States (n = 58), UK (n = 38), India (n = 13), or Thailand (n = 7) and who were in a couple relationship at the time of the surrogacy. Because surrogacy is practiced in similar ways in Thailand and India, with the clinic or agency mediating contact between the surrogate and intended parents, data from participants for these two groups were combined. Ethical approval for the study was granted by the University of Cambridge Psychology Research Ethics Committee.

### Measures

The survey included open-ended and multiple-choice questions about the relationship with the surrogate during pregnancy and after the birth. The questions were based on previous studies of parents' relationships with their surrogates and disclosure patterns 3, 4, 14. The survey was piloted by potential participants to check survey length and functionality and to ensure the questions were meaningful. Data were obtained on [1] how participants had found their surrogate, including whether she was previously known (i.e., a family member/friend) or unknown, and if unknown, whether they had found her through an agency, clinic, website, or other means. Data were collected on whether participants had thought it was important for their surrogate to have particular traits or characteristics, and if yes, which characteristics had been important. Data were also obtained on [2] experiences during pregnancy, including who had updated the participants about the pregnancy, how involved they had felt, and whether they had been happy with their level of involvement. Data were obtained on [3] participants' contact and relationship with the surrogate, including whether they had planned to have contact with her after the birth, whether they currently had contact, and if yes, the frequency of contact, how they maintained contact, and their current relationship with the surrogate (data for these latter two variables were obtained with open-ended questions). Finally, participants were asked whether they had told or planned to tell their child about their birth using surrogacy and (where applicable) egg donation, and their reasons for disclosure (obtained with an open-ended question).

### Analytical Approach

Participants' responses to all multiple-choice questions are reported as number of cases and percentages. Responses to open-ended questions were analyzed using a content analysis approach (15) whereby categories are derived directly from the text data. This approach allows frequency counts to be calculated for subsequent group comparisons. Chi-squared tests, Fisher's exact tests, *t* tests, and one-way analyses of variance were computed to compare differences by location (UK, United States, and Asia) and sexual orientation (heterosexual and gay couples).

### Participants

The majority (66%, 76) of participants were in a heterosexual couple relationship at the time of the surrogacy arrangement, and 34% (40) were in a gay couple relationship. There was no difference between heterosexual and gay couples in where the surrogacy arrangement was conducted, although within Asia all of the gay couples had completed their surrogacy arrangements in Thailand.

Participant characteristics are shown in Table 1. There was a significant association between household income and surrogacy location (Fishers' exact = 0.008), with participants who had U.S. surrogacy having higher incomes. There was no difference by sexual orientation. There was a significant association between type of surrogacy and location of surrogacy (Fisher's exact = 0.000), with intended mothers more likely to have used their own egg for surrogacy arrangements in the UK and all six traditional surrogacy arrangements (i.e. where the surrogate's egg was used) having taken place in the UK. There was also a significant association between type of surrogacy and sexual orientation of the couple (Fishers exact = 0.000), reflecting the greater use of donor eggs by gay couples. The age of the eldest child born using surrogacy ranged from 0 to 11 years (mean = 2.5 years, median = 2 years). Seventy-five percent of children were aged <4 years, with 41% aged <2 years. Heterosexual couples had older children (mean = 2.86 years, SD = 2.47 years) compared with gay couples (mean = 1.88 years, SD = 1.62 years) [*t*(106) = −2.54, *P*=.012].

**Table 1:** Sample characteristics.

Characteristic	UK		United States		Asia	
	Gay couple (n = 12)	Heterosexual couple (n = 26)	Gay couple (n = 23)	Heterosexual couple (n = 35)	Gay couple (n = 5)	Heterosexual couple (n = 15)
Age of participant (y)	42.2 (6.2)	42.1 (6.4)	43.5 (5.6)	45.2 (6.8)	44.4 (6.9)	44.2 (6.2)
Age of child (y)	1.4 (1.4)	3.0 (2.4)	2.0 (1.8)	2.9 (2.8)	2.2 (.44)	2.3 (1.5)
Sex						
Male	12 (100)	2 (7.7)	23 (100)	9 (25.6)	5 (100)	6 (40)
Female	0 (0)	24 (92.3)	0 (0)	26 (74.3)	0 (0)	9 (60)
Ethnicity of respondent						
White	11 (92)	24 (92)	21 (91)	33 (94)	3 (60)	10 (67)
Black	1 (8)	0 (0)	0 (0)	0 (0)	0 (0)	0 (0)
Asian	0 (0)	2 (8)	0 (0)	1 (3)	1 (20)	4 (26)
Mixed	0 (0)	0 (0)	1 (5)	1 (3)	0 (0)	0 (0)
Other	0 (0)	0 (0)	1 (5)	0 (0)	1 (20)	1 (7)
Total household income (£)						
Less than 10,000	0 (0)	1 (4)	0 (0)	0 (0)	0 (0)	0 (0)
10,000–49,999	0 (0)	3 (12)	0 (0)	2 (6)	0 (0)	2 (13)
50,000–99,999	2 (17)	5 (19)	1 (4)	4 (11)	2 (40)	2 (13)
100,000–199,999	7 (58)	9 (35)	6 (26)	11 (31)	2 (40)	6 (40)
200,000–299,999	1 (8)	5 (19)	6 (26)	3 (9)	1 (20)	2 (13)
300,000 or more	2 (17)	1 (4)	9 (39)	14 (40)	0 (0)	1 (7)
Not provided	0 (0)	2 (8)	1 (4)	1 (3)	0 (0)	2 (13)
Type of surrogacy						
Gestational–donor egg	9 (75)	6 (23)	23 (100)	27 (77)	5 (100)	10 (67)
Gestational–intended mother's egg	–	17 (65)	–	8 (23)	–	5 (33)
Traditional	3 (25)	3 (12)	0 (0)	0 (0)	0 (0)	0 (0)

*Note:* Values are mean (SD) or number (percentage).

## Results

### Choosing a Surrogate

Table 2 shows how couples had met their surrogate. Comparisons by location found a significant difference (Fisher's exact = 0.000), showing that participants who had surrogacy in the UK were more likely to have a surrogate who was previously known to them than couples who had surrogacy in other countries. Comparisons by sexual orientation found no differences between gay couples and heterosexual couples in whether their surrogate was previously known.

**Table 2:** How couples met surrogate, important characteristics, and involvement in pregnancy by location and sexual orientation.

Parameter	UK		United States		Asia	
	Gay couple	Heterosexual couple	Gay couple	Heterosexual couple	Gay couple	Heterosexual couple
Met surrogate^a^						
Previously known						
I already knew her, she is a family member	1 (8)	2 (8)	0 (0)	1 (3)	0 (0)	0 (0)
I already knew her, she is a friend	2 (17)	8 (31)	0 (0)	0 (0)	0 (0)	0 (0)
Total	3 (25)	10 (39)	0 (0)	1 (3)	0 (0)	0 (0)
Previously unknown						
Through an agency	4 (33)	11 (43)	22 (96)	33 (94)	4 (80)	5 (33)
Through a clinic	0 (0)	0 (0)	0 (0)	1 (3)	1 (10)	10 (67)
Through a website	3 (25)	2 (8)	0 (0)	0 (0)	0 (0)	0 (0)
Other, please specify (e.g., met through a mutual friend)	2 (17)	3 (12)	1 (4)	0 (0)	0 (0)	0 (0)
Total	9 (75)	16 (63)	23 (100)	34 (97)	5 (100)	15 (100)
Important characteristics^b^						
Education	2 (17)	3 (11)	5 (22)	6 (17)	1 (20)	1 (7)
Medical history	5 (42)	12 (46)	12 (52)	23 (66)	3 (60)	7 (47)
Personality	6 (50)	10 (38)	17 (74)	21 (60)	1 (20)	0 (0)
Physical appearance	2 (17)	2 (77)	4 (17)	3 (9)	0 (0)	4 (27)
Prior experience of surrogacy	1 (8)	5 (19)	4 (17)	7 (20)	2 (40)	1 (7)
Marital status	2 (17)	5 (19)	14 (61)	14 (40)	4 (80)	7 (47)
Other	3 (25)	9 (35)	4 (17)	13 (37)	2 (40)	0 (0)
Who updated parents about the pregnancy						
Surrogate	9 (75)	26 (100)	23 (100)	32 (91)	1 (20)	0 (0)
Clinic	1 (8)	3 (12)	18 (78)	16 (46)	4 (80)	14 (93)
Agency	0 (0)	1 (4)	12 (5)	18 (51)	4 (80)	2 (13)
No one	1 (8)	0 (0)	0 (0)	0 (0)	0 (0)	0 (0)
Other (e.g., medical professionals, attending appointment)	5 (42)	4 (15)	2 (9)	4 (11)	0 (0)	2 (13)
How involved did parents feel in the pregnancy						
Very involved	11 (92)	22 (85)	19 (93)	30 (86)	1 (20)	3 (20)
A little involved	1 (8)	4 (15)	4 (17)	5 (14)	4 (80)	10 (67)
Not involved	0 (0)	0 (0)	0 (0)	0 (0)	0 (0)	2 (13)
Happy with level of involvement						
Yes, very happy	11 (92)	22 (85)	20 (87)	32 (91)	2 (40)	6 (40)
Yes, somewhat happy	1 (8)	1 (4)	3 (13)	3 (9)	2 (40)	8 (53)
No	0 (0)	3 (12)	0 (0)	0 (0)	1 (20)	1 (7)

*Note:* Values are number (percentage).

a Categories were collapsed to previously known vs. previously unknown to run Fisher's exact tests.

b Percentage of those who answered yes, it was important for their surrogate to have particular traits/characteristics.

A significant association was found between whether parents thought it important for their surrogate to have particular characteristics and surrogacy location, with parents who had U.S. surrogacy (88%, 51) seeing this as more important than parents who had undertaken surrogacy in the UK (63%, 24) and Asia (55%, 11) [χ^2^(2) = 11.97, *P*=.003]. The difference by sexual orientation approached significance, suggesting that gay couples were more likely than heterosexual couples to think it was important for their surrogate to have particular characteristics (gay couples = 85%, 35; heterosexual couples 68%, 52) [χ^2^(1) = 3.76, *P*=.07].

Table 2 shows which particular characteristics were reported as important. Personality was seen as more important by couples who had surrogacy in the United States [χ^2^(2) = 22.48, *P*<.001], and marital status was more important for those who had completed their surrogacy in Asia [χ^2^(2) = 10.93, *P*=.004]. No other significant associations were found.

### Experiences During Pregnancy

There was a significant difference between locations in who provided updates on the pregnancy. Those with surrogates in the UK and United States were more likely to be updated by the surrogate compared with those whose surrogate was in Asia (Fisher's exact = 0.000). Couples whose surrogacy had taken place in Asia were more likely to be updated by the clinic [χ^2^(2) = 38.12, *P*<.001], and those who had U.S. surrogacy were more likely to receive updates from the agency [χ^2^(2) = 25.5, *P*<.001] (Table 2).

A significant difference was found between locations in how involved couples felt in the pregnancy. Those in the UK and United States were more likely to report feeling very involved compared with those in Asia (Fisher's exact = 0.000). There were no differences by sexual orientation in how involved couples felt. Participants with United States and UK surrogacy arrangements were more likely to be very happy with their level of involvement compared with those who had surrogacy in Asia, who said they were somewhat happy (Fisher's exact = 0.000).

In terms of whether couples planned to be in contact with the surrogate after the birth, parents whose surrogacy was carried out in Asia were less likely to plan to have contact with the surrogate after the birth compared with couples who had U.S. or UK surrogacy (Fisher's exact = 0.000) and were also less likely to have any current contact (Fisher's exact = 0.000) (Table 3). Parents whose surrogate lived in Asia were in less frequent contact compared with those in the UK and United States (Fisher's exact = 0.04). For parents who had surrogacy in Asia, reasons for lack of contact included communication difficulties, for example, “our surrogate does not speak any English and we do not speak Hindi—this makes having a relationship very difficult,” or that their contact was mediated via the clinic. There was no difference in frequency of contact by sexual orientation.

**Table 3:** Frequency, type of contact, and disclosure decisions by location and sexual orientation.

Parameter	UK		United States		Asia	
	Gay couple	Heterosexual couple	Gay couple	Heterosexual couple	Gay couple	Heterosexual couple
Plan to have contact						
Yes	12 (100)	26 (100)	23 (100)	32 (91)	3 (60)	5 (33)
No	0 (0)	0 (0)	0 (0)	2 (6)	2 (40)	9 (60)
Not provided	0 (0)	0 (0)	0 (0)	1 (3)	0 (0)	1 (7)
Current contact with surrogate (including indirect contact)						
Yes	12 (100)	24 (92)	22 (96)	34 (97)	4 (80)	2 (14)
No	0 (0)	2 (8)	1 (4)	1 (3)	1 (20)	12 (86)
Frequency of current contact?^a^						
Weekly	6 (50)	9 (41)	2 (9)	7 (21)	0 (0)	0 (0)
Monthly	3 (25)	7 (29)	12 (55)	10 (29)	0 (0)	1 (50)
Once every 3 mo	1 (8)	4 (17)	5 (23)	8 (24)	2 (50)	0 (0)
Once or twice per year	2 (17)	4 (17)	3 (14)	9 (26)	2 (50)	1 (50)
Telling: surrogacy^b^						
Yes–told	1 (8)	16 (61)	8 (35)	15 (43)	1 (20)	4 (27)
Yes–plan to tell	11 (92)	9 (35)	15 (65)	19 (54)	4 (80)	10 (67)
Undecided	0 (0)	1 (4)	0 (0)	1 (3)	0 (0)	1 (6)
Not planning to tell	0 (0)	0 (0)	0 (0)	0 (0)	0 (0)	0 (0)
Telling: egg donation^c^						
Yes–told	1 (10)	0 (0)	4 (17)	10 (37)	1 (20)	1 (10)
Yes–plan to tell	8 (80)	6 (86)	19 (83)	13 (48)	3 (60)	7 (70)
Undecided	1 (10)	0 (0)	0 (0)	1 (4)	1 (20)	2 (20)
Not planning to tell	0 (0)	1 (14)	0 (0)	3 (11)	0 (0)	0 (0)
Reasons for telling						
Child's right to know	1 (10)	5 (24)	0 (0)	6 (19)	0 (0)	3 (27)
Family structure	4 (40)	0 (0)	8 (40)	0 (0)	1 (20)	0 (0)
Importance of honesty	5 (50)	10 (48)	7 (35)	17 (53)	3 (60)	3 (27)
Ongoing relationship with surrogate	3 (30)	1 (5)	3 (15)	2 (6)	0 (0)	0 (0)
Proud of conception	2 (20)	16 (76)	3 (15)	8 (25)	0 (0)	5 (45)
Others know	0 (0)	1 (5)	0 (0)	5 (16)	0 (0)	0 (0)
Important child knows their story/identity	0 (0)	1 (5)	8 (38)	5 (16)	1 (20)	3 (27)
Advised to tell	1 (10)	1 (5)	0 (0)	4 (13)	0 (0)	0 (0)

*Note:* Values are number (percentage).

a For those in contact.

b Categories were collapsed into told/plan to tell vs. undecided/not telling to run χ^2^ tests.

c For those who used egg donation.

### Comparisons Between UK and United States for Parents' Current Relationship With the Surrogate

Because parents who had surrogacy in Asia were less likely to have direct contact with their surrogate compared with those in the United States and UK, the following analyses compare participants who had undertaken surrogacy in the UK and United States only.

Table 4 shows the categories for parents' description of their current relationship with the surrogate. There was no difference between heterosexual and gay couples in the way in which the relationship with the surrogate was described, and no difference was found between the UK and United States. When previously known surrogates (n = 14) were removed, both comparisons remained nonsignificant.

**Table 4:** Quality of relationship with surrogate for parents who had completed their surrogacy in the UK and United States.

Quality	UK		United States	
	Gay couple	Heterosexual couple	Gay couple	Heterosexual couple
Very positive	7 (58)	11 (44)	9 (39)	15 (44)
Positive	3 (25)	10 (40)	9 (39)	10 (29)
Neutral	2 (17)	2 (8)	2 (9)	7 (20)
Distant	0 (0)	0 (0)	2 (9)	2 (6)
None	0 (0)	2 (8)	1 (4)	0 (0)

*Note:* Values are number (percentage). Categories were collapsed to very positive/positive vs. neutral/distant/none to run χ^2^ tests.

Among those who had a “very positive” relationship with their surrogate were those who viewed their surrogate as family or “like family,” as “closest of friends” or “best friend,” and those who described their relationship as “excellent,” “very strong,” or “very warm.” Descriptions from families included statements such as, “she is our son's godparent, and we would consider her part of our family,” or, “I feel like she is a friend with whom I have had a very emotional relationship and we therefore have a close bond,” or, “she and her family became like family to us whilst we stayed in California.”

Parents who had a “positive” relationship with their surrogate described it as “good,” “amicable,” “trusting,” or simply “friendly.” As one parent said, “(the relationship is) friendly—we are all just getting on with our lives.”

A smaller number of families described their relationship with their surrogate as “casual,” “cordial,” or other terms implying a more neutral affect and relationship, for example, “we're in touch every now and then to say hi.”

In four cases the relationship was categorized as “distant.” In two of these families the relationship had changed since the surrogacy, and the parents described challenges in the relationship: “our surrogate avoided us for about a year after the first year. We could not quite understand it. However, she regretted the distance she had created and wanted to rekindle the friendship. This has not been so easy. So it is more distant than it used to be.”

In terms of how contact was maintained between parents and surrogates, there were no differences between couples who had surrogacy in the UK or United States in their likelihood of keeping in contact via social media, messaging/e-mail, calling, or sending cards, but couples who had UK surrogacy were more likely to maintain contact through visiting their surrogate than those who had had U.S. surrogacy [χ^2^(1) = 15.18, *P*<.001]. There were no differences between heterosexual and gay couples in their methods of keeping in contact with their surrogate.

### Telling the Child About Surrogacy and Egg Donation

There was a significant difference between heterosexual and gay couples in telling the child about their surrogacy conception (Fisher's exact = 0.03), which reflected three heterosexual couples who were undecided in whether they would tell. There was no difference in couples' decisions about whether to tell the child according to the country in which the surrogacy had taken place. Whether couples were in contact with the surrogate or type of surrogacy was also not related to parents' decision to tell their child.

For couples who had used donor eggs or the surrogates egg (as oppose to the intended mothers egg), there were no differences in couples' decisions about whether to tell the child about the use of a donor egg according to either sexual orientation of the couple or location of the surrogacy.

Table 3 shows parents' reasons for telling the child about their surrogacy conception. Only one association between country and reasons cited was significant, with couples who had surrogacy in the UK more likely than couples who had surrogacy elsewhere to say that they planned to tell the child because they were proud of their conception story (Fisher's exact = 0.02). Couples who had US surrogacy were marginally more likely to say that they planned to tell their child because surrogacy was part of the child's story/important for the child's sense of identity (Fisher's exact = 0.06). Gay couples were more likely than heterosexual couples to cite their family structure as a reason to tell their child (Fisher's exact = 0.000), and heterosexual couples were more likely to cite pride in their conception story as a reason for telling [*χ*^2^(1) = 5.89, *P*=.02].

## Discussion

The findings from this study highlight the similarities and differences in UK parents' relationships with their surrogates during pregnancy and after birth, depending on the country in which the surrogacy was carried out. Overall, relationships between parents and surrogates were largely similar between those who had surrogacy in the UK and United States and differed most with Asia. Thus the country to which couples traveled for surrogacy affected parents' level of involvement in the pregnancy and their subsequent relationship with the surrogate rather than travelling overseas per se.

Parents who had surrogacy in the United States were more likely to think it important that their surrogate had particular traits and characteristics. This finding reflects the marketization of U.S. surrogacy and the greater choice of available surrogates, as compared with the UK, which currently has a shortage of surrogates, and Asia, where surrogates often remain anonymous to the intended parent. Parents who had surrogacy in Asia were more likely to view marital status as an important characteristic that is necessary for obtaining legal parenthood via a parental order in the UK.

Parents who had surrogacy in Asia were less likely to have any direct contact with the surrogate and generally felt uninvolved in the pregnancy. They were also less likely to intend to be in contact with the surrogate after the birth and were indeed in less contact currently. This lack of contact, although preferred by some couples, was a result of the way in which surrogacy was managed, that is, with parents having a relationship with the clinic rather than the surrogate during the pregnancy. In addition, the language differences hindered any attempts for direct communication. It is no longer possible for foreigners to access surrogacy in India and Thailand; however, other countries that follow similar models of surrogacy (i.e., clinic-mediated contact) and where language differences exist are also likely to result in distant relationships with the surrogate. Indeed, data from the larger study from which this sample is drawn found that intending parents were considering surrogacy in countries such as Georgia and Ukraine, where they may also face similar communication difficulties with the surrogate (16). Lack of contact might lead to stronger relationships within the family unit because the involvement of the surrogate would not interfere in parent–child relationships (17); however, it is also possible that the child might be curious about their surrogate or want to meet her in the future (18), which would be particularly difficult in cases in which the surrogate was anonymous to the parents.

Parents who had surrogacy in the United States and UK felt involved in the pregnancy and did not differ in their descriptions of their current relationships with the surrogate, with most couples describing it as positive or very positive. Given the available technologies that now exist to maintain indirect contact between people residing in different countries (and the effect this has had on the ways in which relationships are maintained [19]), it is perhaps unsurprising that close relationships exist between couples and U.S. surrogates. It is important to note that surrogacy in the United States can be costly, and indeed couples who carried out U.S. surrogacy had higher family incomes than those who had surrogacy in Asia and UK. Given the disparities in couples' experiences of surrogacy by country, more expensive destinations such as the United States that enable greater involvement with the pregnancy and surrogate may be unaffordable to many couples planning to use surrogacy. Staying in the UK is also not an option for all couples, given the shortage of surrogates in the UK and the delay in finding a surrogate. However, it is important to note that parents who travelled to Asia for surrogacy were less likely to expect a relationship with the surrogate after the birth and thus may have chosen a destination that would facilitate lack of contact.

Most parents in this study had children under the age of 4 years. Previous studies have found that frequency of contact lessens over time 4, 20 and can differ between traditional and gestational surrogates, and previously known and previously unknown surrogates, with less frequent contact maintained with surrogates who were traditional surrogates and those who were previously unknown to the couple (4). In the present study it was not possible to compare the experiences of traditional to gestational surrogates because only six couples had used traditional surrogacy. Parents who had UK surrogates were more likely to have known their surrogate before the surrogacy compared with parents who had traveled abroad, and thus it would be expected that they may be more likely to maintain future contact.

Because of the lack of commercial surrogacy agencies in the UK, intended parents and surrogates often have to work closely together during the pregnancy, which can lead to strong relationships forming (21). However, not all couples and surrogates agree to continue contact after the birth of the child (5), and the frequency of contact has not been found to be related to how surrogates, parents, or children feel about each other or about the surrogacy arrangement 4, 12. That is, those involved in surrogacy can still feel positive about the surrogacy regardless of whether they have contact with each other. Dissatisfaction among surrogates was found when an expectation to maintain contact after the birth was not met by couples (5).

This was the first study to compare the experiences of gay and heterosexual couples who had used surrogacy to have their child. Differences were found in whether parents planned to tell their child about their surrogacy birth, with all gay couples planning to tell, or having told, their child(ren) and three heterosexual couples being undecided. Studies have found that heterosexual couples using surrogacy are much more likely to tell their child about surrogacy compared with heterosexual couples using other forms of third-party reproduction (e.g., egg or sperm donation) (22). With regard to reasons for telling, that UK couples were more likely than couples who had traveled abroad to cite pride in their conception story as a reason may be partly explained by the surrogacy process in the UK, where surrogacy takes place in a relatively small community in which close relationships are encouraged. That gay couples were more likely to cite family structure as a reason mirrors findings in a study of U.S. gay fathers through surrogacy, in which most fathers who had started the disclosure process did so by explaining to their children that two men needed help in creating a family (3).

This study found that sexual orientation was not an important factor in determining the type of relationships couples have with their surrogate during pregnancy and after the birth. Counsellors should guard against making assumptions based on sexual orientation and should instead focus on the practical features of surrogacy (e.g., direct contact with surrogate, ease of communication) that might influence the parent–surrogate relationship. Counsellors can also highlight how the way in which surrogacy is practiced in different countries might affect how involved parents feel in the pregnancy and also discuss the implications of future contact with the surrogate.

Children aged 4–12 years born through surrogacy to Italian gay couples have been reported to feel indifferent/uninterested in their birth and to show more interest in their surrogate than their egg donor (6). Adolescents born using surrogacy have been reported to feel either positive or indifferent about their surrogacy birth at age 14 years (18). However, this latter study found that approximately half of the adolescents who had no contact with their surrogate were interested in her, with the remainder being uninterested. Although the numbers are small, these findings suggest that some children born through surrogacy may have questions about their surrogate in the future or may express a desire to meet her. It is not known how the lack of information that some couples in the present study had about their surrogate will affect children in the future. It is possible that children in gay couple families may differ from those in heterosexual couple families in their curiosity about the surrogate or donor. In a study of adults requesting information about their sperm donor's identity, it was found that approximately one-quarter of adults from heterosexual couple families requested information about their donor, compared with approximately one-third of adults from lesbian couple families and more than half from single-mother families (23). It has also been found that children who are securely attached to their parents are more likely to be curious about their sperm donor (24), reinforcing the need for more detailed longitudinal studies of parenting and child outcome in families formed through different surrogacy practices.

The present study has a number of limitations. The representativeness of the sample is unknown, and the survey methodology did not enable in-depth exploration of couples' relationships with their surrogate. Recruitment through the law firm and surrogacy organization enabled access to couples who had carried out surrogacy in the UK and overseas. However, the sample may not be representative of all couples who have used surrogacy. Furthermore, not all couples who travel overseas for surrogacy seek professional assistance, and it is possible that couples in these arrangements have different experiences from those who seek advice from professional organizations.

In conclusion, findings from this study show that parents' surrogacy experiences during and after the pregnancy vary according to the location of the surrogacy arrangement. Gay and heterosexual couples from the UK who have surrogacy in the UK and United States maintained positive relationships with the surrogate during and after the birth of the child. Couples who had surrogacy in Asia were less likely to plan to be in contact after the birth, suggesting that destinations that do not facilitate direct contact with the surrogate might be preferred by those who do not wish to maintain a relationship. Future research should examine what these differences mean for families as the children grow up.
